# Confirming the association between low serum 25OHD levels in girls with central precocious puberty and its severity

**DOI:** 10.1186/s12887-023-04447-7

**Published:** 2023-12-09

**Authors:** Liya Xu, Pin Li, Dandan Yuan

**Affiliations:** Department of Pediatric Endocrinology, Shanghai Children’s Hospital, School of medicine, Shanghai Jiao Tong University, Shanghai, 200062 China

**Keywords:** Central precocious puberty (CPP), Vitamin D deficiency, 25-hydroxyvitamin D (25OHD

## Abstract

**Background:**

To assess the differences in vitamin D levels in girls with rapidly progressive (RP) or slowly progressive (SP) central precocious puberty (CPP) and to compare whether the factors related to RP-CPP influenced the vitamin D status. A cross-sectional study was performed among girls with CPP classified as RP-CPP or SP-CPP.

**Methods:**

The baseline data, gonadotropin-releasing hormone (GnRH) stimulation test results, serum 25-hydroxyvitamin D (25OHD) levels, and season of sample collection were analyzed.

**Results:**

The mean 25OHD level in 340 girls was 15.89 ± 6.87 ng/mL, of whom only 10 (2.9%) had normal levels (≥ 30 ng/mL). A total of 114 girls in the SP-CPP group and 226 in the RP-CPP group had similar chronological age, disease course, height SDS, bone mineral density, baseline follicle-stimulating hormone (FSH), peak FSH, and 25OHD levels. Developmental age, body mass index (BMI), BMI SDS, peak luteinizing hormone (LH)/FSH, insulin-like growth factor 1 (IGF-1), and IGF-1 SDS were independent risk factors for RP-CPP. Significant differences were observed among the different serum 25OHD levels in terms of season, disease course, IGF1 level, and BMI SDS (P < 0.05). Moreover, the sampling season was strongly correlated with serum 25OHD levels (r = 0.402, P < 0.001).

**Conclusion:**

The vitamin D levels were generally deficient or insufficient in girls with CPP, but were not related to the different types of CPP. High BMI levels, IGF1 levels, or peak LH/FSH ratio, but not vitamin D levels, could promote the progression of RP-CPP. Seasonal factors mainly influenced the vitamin D levels.

## Background

The development of secondary sex characteristics before the age of 8 years in girls or before the age of 9 years in boys is considered precocious puberty, which is relatively common in girls [1–3]. Central precocious puberty (CPP) and peripheral precocious puberty (PPP) are distinguished by whether the condition resulted from the central activation of the hypothalamic-pituitary-gonadal axis (HPGA), which is the case in CPP [3]. The prevalence of CPP has been increasing in recent years and varies across countries [4, 5]. CPP in girls is of particular concern as it may result in early menarche, reduced adult height, and psychological problems [6–8]. These problems will more likely occur in girls with rapidly progressive CPP (RP-CPP), in which the rate of pubertal development is more accelerated and proceeds much faster compared with that of normal puberty [2, 7]. A growth spurt is an invariably characteristic manifestation of RP-CPP [7]. In contrast, slowly progressive CPP (SP-CPP) is slower and considered to be fairly benign even without treatment [9, 10], which is usually accompanied by a relatively low growth velocity [3, 10].

Vitamin D deficiency is one of the most common diseases worldwide to date [11]. The prevalence of vitamin D deficiency and insufficiency, assessed based on the serum 25OHD levels, is relatively high in children and adolescents [12]. Vitamin D plays an important role in skeletal metabolism; multiple studies have demonstrated many extra-skeletal actions of vitamin D in different diseases such as cancer, neurologic diseases, diabetes and metabolic syndrome, cardiovascular diseases, immunological diseases, et al. [13]. However, only a few studies have investigated its action in the reproductive system. Several recent studies have shown that children with vitamin D deficiency are more likely to develop precocious puberty [9, 14, 15]. Several studies have shown that girls deficient in vitamin D had a higher risk of early menarche than girls sufficient in vitamin D [16, 17]. Previous studies have reported that the serum vitamin D levels in CPP girls were lower than those in healthy girls [9, 15]; however, without classifying it as RP-CPP or SP-CPP, little is known about the difference in vitamin D levels between the two CPP types, as well as the risk of RP-CPP in girls, who demonstrate an accelerated linear growth. Whether adequate vitamin D levels are necessary for girls with RP-CPP remain unknown.

Therefore, this study aimed to assess the differences in serum vitamin D levels in girls with different types of CPP and to investigate the risk of RP-CPP, as well as other indicators.

## Methods

### Patients

This cross-sectional study included girls with CPP who were diagnosed or treated at the Shanghai Children’s Hospital between April 2017 and April 2019.

The CPP girls enrolled in this study were aged 6–10 years. Girls (1) with secondary sex characteristics that appeared before the age of 8 years, (2) whose gonadotropin-releasing hormone (GnRH) stimulation test results showed peak stimulated luteinizing hormone (LH) level of ≥ 5 U/L, and (3) with an ovarian volume of ≥ 1 ml were included in this study [2, 5].

Girls (1) with slow annual growth (< 5 cm/year), (2) with CPP who had previously received drug therapy for precocious puberty, (3) with secondary CPP induced by other causes, (4) who had been supplemented with vitamin D prior to the study enrollment, and (5) who had unavailable clinical data were excluded.

This study was approved by the ethics committee of our hospital. Informed consent was obtained from all patients and their guardians.

### Grouping

Girls with CPP were grouped according to CPP type. Girls with at least one of the following criteria were considered to have RP-CPP and were placed in the RP-CPP group: (1) showed progression of breast development from one Tanner stage to the next within 3–6 months. (2) demonstrated a linear growth (height increased by > 3 cm within 6 months); and (3) an advanced bone age (BA) of at least 1 year [2, 7]. Otherwise, the girls were considered to have SP-CPP and were assigned to the SP-CPP group. Overall, the main distinguishing factors between the two groups were the rate of progression of physical findings and bone maturation.

The 25OHD levels were divided into four groups according to the following threshold levels: <10 ng/mL, 10 ng/mL ≤ 25OHD < 15 ng/mL, 15 ng/mL ≤ 25OHD < 30 ng/mL, and ≥ 30 ng/mL were categorized as severe deficiency, deficiency, insufficiency, and normal vitamin D levels, respectively.

The year was divided into two periods: the winter-spring season (November to April) and summer-autumn season (May–October) [19].

### Data collection and measurements

The baseline data of the girls, including their age, height, weight, and body mass index (BMI), were collected. All the girls underwent a GnRH stimulation test. This test involved intravenous injection of gonadorelin at a dose of 2.5 µg/kg (with the maximum total dose of 100 µg), and the serum levels of LH and FSH were measured at 0, 30, 60, and 90 min after injection using a chemiluminescence immunoassay (UniCel DxI 800 Access Immunoassay System, Beckman Coulter, USA). Bone mineral density (BMD) was measured using dual X-ray bone densitometry (EXA-3000; OsteoSys, Korea). BA was measured by Greulich and Pyle’s standards based on left hand radiograph [17]. An electrochemiluminescence binding assay was performed to measure the 25OHD levels (cobas e601, Roche Diagnostics, Germany).

### Statistical analysis

SPSS (version 23.0; IBM Corp., USA) was used to perform all statistical analyses. A normality test was conducted for all continuous data. For data with a normal distribution, means and standard deviation (SD) were used for the description, and an independent t-test was used for comparisons. For data with non-normal distribution, median (M) and percentiles (P25 and P75) were used for the description, and the Mann–Whitney U test was used for comparisons. Multivariate logistic regression was used for the analysis of factors associated with RP-CPP, and the indicators with a P value of < 0.05 in the univariate regression analysis were included. The Pearson’s (for continuous data with a normal distribution) or Spearman’s (for categorical data or continuous data with non-normal distribution) correlation coefficient tests were used to examine the correlation of the indicators with 25OHD. P values < 0.05 were considered significant.

## Results

### Baseline data of patients

In total, 340 girls with CPP were included in this study. Of them, 114 (33.5%) were in the SP-CPP group and 226 (66.5%) were in the RP-CPP group. A total of 226 girls were in the RP-CPP group, of whom only 2 met criterion 1), 3 only met criterion 2), 74 met criteria (1) and 3), and 85 met criteria (2) and 3), additionally, 62 girls met all three criteria. A comparison of the baseline data between the two groups is presented in Table 1. The chronological age(CA) of the girls was similar between the two groups with median ages of 8.42 (7.90–8.92) years in the SP-CPP group and 8.50 (8.17–8.92) years in the RP-CPP group. Moreover, the 25OHD level was similar in both groups (P = 0.369). Significant differences were observed in the developmental age (P = 0.022), BA (P < 0.001), height(P = 0.009), weight (P = 0.002), BMI (P = 0.007), BMI SDS (P = 0.010), peak LH/FSH ratio (P < 0.001), IGF-1 level (P < 0.001), IGF1 SDS (P < 0.001), baseline LH level (P < 0.001), peak LH level (P < 0.001), advanced bone age (P < 0.001) and Tanner stage of breast development (P < 0.001) between the two groups. However, height SDS was similar in both groups (P = 0.074).

**Table 1:** Baseline data of girls with CPP

Variable	SP-CPP (n = 114)	RP-CPP (n = 226)	P
CA (years)	8.42 (7.90–8.92)	8.50 (8.17–8.92)	0.375
Developmental age (years)	7.00 (6.33–7.52)	7.25 (6.50–7.75)	0.022
BA (years)	9.29 (8.83–10.00)	10.00(9.83–11.00)	< 0.001
Disease course (years)	1.25 (0.89–2.00)	1.25 (0.92–2.00)	0.730
Height(cm)	135.05(130.75–138.03)	137.00(132.00–141.05)	0.009
Weight(kg)	30.00(27.10–34.10)	32.00(28.71–36.46)	0.002
BMI (kg/m^2^)	16.50 (15.66–17.79)	17.37 (15.87–18.76)	0.007
Height SDS	1.00(0.31–1.87)	1.35 (0.45–2.20)	0.074
BMI SDS	0.26(-0.08–0.73)	0.4954(0.04–0.99)	0.010
IGF1 SDS	0.54(0.13–1.03)	1.1352(0.57–1.62)	< 0.001
25OHD (ng/ml)	16.10 ± 6.84	15.78 ± 6.91	0.369
Peak LH/FSH ratio	0.76 (0.48–1.38)	1.42 (1.00–2.02)	< 0.001
BMD	0.10 (− 0.30–0.60)	0.20 ((− 0.20–0.70)	0.207
IGF-1 level (ng/ml)	287.00 (220.50–346.50)	358.50 (291.75–419.00)	< 0.001
Baseline LH level (IU/L)	0.72 (0.36–1.43)	1.12 (0.58–2.11)	< 0.001
Peak LH level (IU/L)	9.19 (6.54–20.27)	20.89 (11.21–30.41)	< 0.001
Baseline FSH level (IU/L)	4.73 ± 2.34	4.46 ± 2.20	0.483
Peak FSH level (IU/L)	13.35 (10.52–19.96)	13.15 (10.72–17.41)	0.903
Advanced bone age (years)	0.87 (0.48–1.42)	1.75 (1.42–2.08)	< 0.001
Tanner stage of breast development			< 0.001
B2	29 (25.5%)	30 (13.3%)	
B3	73 (64.0%)	164 (72.6%)	
B4	12 (10.5%)	32 (14.1%)	

CPP, central precocious puberty; SP, slowly progressive; RP, rapidly progressive; CA, chronological age; BA, bone age; BMI, body mass index; 25OVHD, 25-hydroxyvitamin D; LH, luteinizing hormone; FSH, follicle-stimulating hormone; BMD, bone mineral density; IGF-1, insulin-like growth factor 1

### Multivariate analysis of the factors associated with RP-CPP

When multivariate analysis was used to determine the factors associated with RP-CPP (Table 2), developmental age (OR = 0.751, 95% confidence interval (CI): 0.570–0.989, P = 0.041), BMI (OR = 2.690, 95% CI: 1.899–3.809, P < 0.001), BMI SDS (OR = 1.689, 95% CI: 1.010–3.424, P = 0.012), IGF-1 (OR = 3.288, 95% CI: 1.540–7.020, P = 0.002), IGF-1 SDS (OR = 2.185, 95% CI: 1.132–4.596, P = 0.006), and peak LH/FSH ratio (OR = 1.005, 95% CI: 1.001–1.008, P = 0.015) were identified as significant independent factors; however, BA, baseline LH, peak LH, and height SDS were not correlated with it.

**Table 2:** Multivariate analysis of the factors related to rapidly progressive CPP

Indicator	OR	95%CI	P
Developmental age	0.751	0.570–0.989	0.041
BMI	2.690	1.899–3.809	< 0.001
BA	1.012	0.881–1.162	0.871
IGF-1	3.288	1.540–7.020	0.002
Peak LH/FSH	1.005	1.001–1.008	0.015
Baseline LH	0.828	0.623–1.099	0.190
Peak LH	0.997	0.960–1.035	0.859
Height SDS	1.040	0.789–1.371	0.781
BMI SDS	1.689	1.010–3.424	0.012
IGF-1 SDS	2.185	1.132–4.596	0.006

CPP, central precocious puberty; BMI, body mass index; BA, bone age; LH, luteinizing hormone; FSH, follicle-stimulating hormone; IGF-1, insulin-like growth factor 1

### 25OHD levels

The mean serum 25OHD level of the CPP girls was 15.89 ± 6.87 ng/ml. Specifically, 68 (19.9%) girls had severely deficient 25OHD, 110 (32.4%) had deficient 25OHD level, and 152 (44.7%) had insufficient 25OHD. Only 10 (2.9%) girls had a normal 25OHD level (> 30 ng/mL). The results of the correlation analysis are shown in Table 3. Chronological age (CA, r = − 0.114, P = 0.035), disease course (r = − 0.114, P = 0.035), peak LH/FSH ratio (r = − 0.110, P = 0.043), BMI (r = − 0.097, P = 0.074), BMI SDS (r = − 0.164, P = 0.027), IGF-1 level (r = − 0.175, P = 0.001), and IGF-1 SDS (r = − 0.109, P = 0.256) were negatively correlated with reduced 25OHD levels. BMD (r = 0.126, P = 0.023) and height SDS (r = 0.079, P = 0.554) was positively correlated, and the season of sample collection (r = 0.402, P < 0.001) was positively associated with reduced 25OHD levels. Among these factors, the season of sample collection (r = 0.402, P < 0.001) was **s**trongly associated with reduced 25OHD levels. More girls had vitamin D deficiency during the winter-spring season (December–May) compared with that during the summer-autumn season.

**Table 3:** Correlation analysis of the factors associated with reduced 25OHD levels in girls with CPP

	Correlation coefficient (r)	P value
CA	−0.114	0.035
Developmental age	−0.021	0.696
Disease course	−0.114	0.035
BA	−0.078	0.153
BMI	−0.097	0.074
Peak LH/ FSH	−0.110	0.043
BMD	0.126	0.023
IGF-1	−0.175	0.001
Baseline LH	−0.084	0.121
Peak LH	−0.044	0.420
Height SDS	0.079	0.554
BMI SDS	-0.164	0.027
IGF-1 SDS	-0.109	0.256
Season	0.402	< 0.001

Abbreviations: CPP, central precocious puberty; CA, chronological age; BA, bone age; BMI, body mass index; LH, luteinizing hormone; FSH, follicle-stimulating hormone; BMD, bone mineral density; IGF-1, insulin-like growth factor 1

## Discussion

The present study has three main findings. First, 340 CPP girls had a high prevalence of vitamin D deficiency. In this study, 178 girls (52.4%) had varying degrees of 25OHD deficiency. However, no difference was observed between the SP-CPP and RP-CPP groups in terms of 25OHD levels. Second, results of the multivariate analysis showed that developmental age, BMI, IGF-1 level, BMI SDS, IGF-1 SDS, and peak LH/FSH ratio were independently associated with RP-CPP, but 25OHD levels were not. Finally, the correlation analysis showed that the season of sample collection was strongly associated with reduced 25OHD levels.

Recently, several clinical trials and meta-analyses have shown that precocious puberty may be associated with vitamin D deficiency [8]. Almost all previous studies revealed that girls with CPP had lower 25OHD levels compared with healthy girls [8, 14, 15]; therefore, a control group was not included in this study, and girls with RP-CPP were compared with those with SP-CPP. These two types of CPP are classified mainly based on the presence or absence of disease progression and have important clinical implications for both treatment and prognosis. Girls with RP-CPP often present with growth spurt and accelerated gonadal organ development. Without treatment, they eventually experience early menarche and short adult stature [2]. Conversely, girls with SP have a relatively slow, benign disease progression with a good prognosis despite the absence of treatment [10]. However, no previous study investigating the vitamin D status in girls with CPP has divided the patients into RP-CPP and SP-CPP, and most previous studies only included girls with RP-CPP [15, 18–21] and omitted those with SP-CPP. In the present study, 52.4% of girls with CPP had 25OHD deficiency, regardless of the CPP type. The 25OHD level in the RP-CPP group was not lower than that in the SP-CPP group, which indicated that low 25OHD levels in girls with CPP were not associated with the severity of CPP. The present study was the first to include girls with SP-CPP and investigated the relationship between different types of CPP and 25OHD levels.

In this study, girls with CPP who had higher BMI, IGF1 level, BMI SDS, IGF-1 SDS, and peak LH/FSH ratio were more likely to have RP-CPP. By making this distinction, we were able to determine whether the factors related to RP-CPP influenced the vitamin D status. CA, disease course, peak LH/FSH ratio, IGF-1 level, BMD, BMI SDS and season of sample collection were correlated with reduced 25OHD levels. Girls with RP-CPP may be at higher risk of experiencing calcium insufficiency due to their rapid growth spurt; therefore, additional vitamin D and calcium should be provided to this patient group. However, the results of this study suggest that faster growth was not an important factor, but the season was. By contrast, this study found an inverse correlation between 25OHD levels and the disease course. A correlation with the LH/FSH ratio was also observed in this study. Previous retrospective studies indicated that pubertal children had lower vitamin D levels compared with prepubertal children [22, 23]. This finding indicated that a reduction in 25OHD levels was related to the high peak LH/FSH ratio, which was also a factor related to the occurrence of RP-CPP. IGF-1 is also associated with the timing of CPP [24] and was correlated with vitamin D level in this study. These results suggest that the relationship between the vitamin D status and CPP requires further investigation. Previous studies have also found a relationship between vitamin D level and the age of children, with a negative correlation between children’s CA and 25OHD level [23, 25, 26]. This study also found a significantly negative correlation between vitamin D level and age. This could be related to the changes in diet and exercise in children as they grow older.

Season is one of the main factors that influence the vitamin D levels, which are lower during spring than during autumn [19, 27]. However, a Romanian study showed that the serum levels of 25OHD had a marked seasonal variation, with the highest levels in September (early autumn) and lowest levels in March (early spring), which persisted in all age and sex groups [28]. The seasonal variation in vitamin D levels is supported by the results of our study and is not surprising because sunlight is one of the main sources of vitamin D. The levels of vitamin D in spring and winter are likely to be lower because girls lack sunlight exposure and spend less time outdoors during colder weather [29]. This finding suggests that vitamin D supplementation during winter and spring may be necessary for girls with CPP.

This study has some limitations. As a cross-sectional study, it could not determine any causal relationship; therefore, further study is needed to determine whether vitamin D deficiency is a result of CPP or may be a mechanism by which CPP occurs in some girls. Since the study was conducted in a single hospital, it had a relatively small sample size; as it was a single-center study, the results may not be applicable to the larger population of China.

## Conclusions

This study showed that girls with CPP were generally vitamin D deficient or insufficient. However, the type of CPP did not influence the vitamin D levels because they were similar in the SP-CPP and RP-CPP groups. Higher BMI, IGF1 levels, and peak LH/FSH ratios may contribute to the progression to RP-CPP. Seasonal factors have a major influence on the vitamin D levels. Vitamin D supplementation should be considered in girls with any type of CPP, both RP-CPP and SP-CPP, especially during the spring and winter months, as they are more likely to experience vitamin D deficiency during these seasons.

## Data Availability

The datasets used and/or analysed during the current study are available from the corresponding author on reasonable request.
